# Evaluation of hGM-CSF/hTNFα surface-modified prostate cancer therapeutic vaccine in the huPBL-SCID chimeric mouse model

**DOI:** 10.1186/s13045-015-0175-8

**Published:** 2015-06-25

**Authors:** Shouhua Lai, Zhiyong Huang, Yunting Guo, Yunqin Cui, Lei Wang, Weifeng Ren, Furong Ying, Hui Gao, Lingxia He, Tieli Zhou, Jiegen Jiang, Jimin Gao

**Affiliations:** Zhejiang Provincial Key Laboratory for Technology and Application of Model Organisms, School of Laboratory Medicine and Life Sciences, Wenzhou Medical University, Wenzhou, Zhejiang 325035 China; Department of Cardiothoracic Surgery, Nanfang Hospital, Southern Medical University, Guangzhou, Guangdong 510515 China

**Keywords:** Cancer immunotherapy, Adjuvant, Synergetic effect, Human immune system, PC-3 prostate cancer cell

## Abstract

To validate its efficacy in the context of the human immune system, a novel therapeutic vaccine of hGM-CSF/hTNFα surface-modified PC-3 cells against human prostate cancer was evaluated in the human peripheral blood lymphocytes-severe combined immunodeficiency (huPBL-SCID) chimeric mouse model. The hGM-CSF or/and hTNFα modified vaccines inhibited prostate cancer growth effectively so as to prolong the mouse survival significantly. The splenocytes from the hGM-CSF/hTNFα vaccine-inoculated mice showed the strongest tumor-specific cytotoxicity against PC-3 cells and the highest production of IFNɤ. These features indicated that type 1 protective immune response was induced efficiently against human prostate cancer and further enhanced through synergetic adjuvant effects of hGM-CSF and hTNFα.

**Electronic supplementary material:**

The online version of this article (doi:10.1186/s13045-015-0175-8) contains supplementary material, which is available to authorized users.

## Findings

Prostate cancer is currently the second leading cause of cancer-related death in elderly men and likely develops into androgen-independent at advanced stages, which is refractory to conventional treatments. Cancer vaccine is a rational option for the treatment of androgen-independent prostate cancer [1]. Cancer immunotherapy is getting more and more important, remarkably evidenced by recent checkpoint blockade or chimeric antigen receptor-engineered T cell-based clinical trials with the impressive efficacy in different types of metastatic cancers [2, 3].

Through simultaneous immobilization of streptavidin-tagged bioactive GM-CSF and TNFα on the biotinylated surface of cancer cells, we previously showed that the resultant cancer vaccines could induce a strong anti-tumor T cell immunity [4–7]. In the current study, human peripheral blood lymphocytes-severe combined immunodeficiency (huPBL-SCID) model was utilized to mimic the human immune system for evaluating the efficacy of these therapeutic vaccines. The materials and methods used in this study are detailed in Additional file 1.

We demonstrated that SA-hGM-CSF or/and SA-hTNFα could be efficiently immobilized on the biotinylated surface of ethanol-fixed PC-3 cells (Additional file 2) and SA-hGM-CSF or/and SA-hTNFα immobilized on the PC-3 cells still retained their bioactivity (Additional file 3).

The PC-3 cells inoculated subcutaneously in nonobese diabetic/severe combined immunodeficiency (NOD/SCID) mice were found to maintain their original tumorigenicity so as to spread into the blood (Additional file 4). There was no phenomenon of immune leakage [8] in NOD/SCID mice used in this study. The activity of natural killer (NK) cells in the NOD/SCID mice was dramatically reduced by injection of anti-asialo-GM1 antibody (Additional file 5).

Flow cytometry detected human CD4^+^, CD8^+^, and CD45^+^ cells in peripheral blood and human CD45^+^ cells in spleen of huPBL-SCID mice. IHC staining revealed many human CD4^+^ and CD8^+^ lymphocytes present in spleen and fewer human CD4+ and CD8+ lymphocytes in liver tissue 8 weeks after transfer of huPBL. The results indicated that human T lymphocytes were successfully engrafted and homed in appropriate lymphoid organs of huPBL-SCID mice (Additional files 6 and 7).

**Fig. 1:**
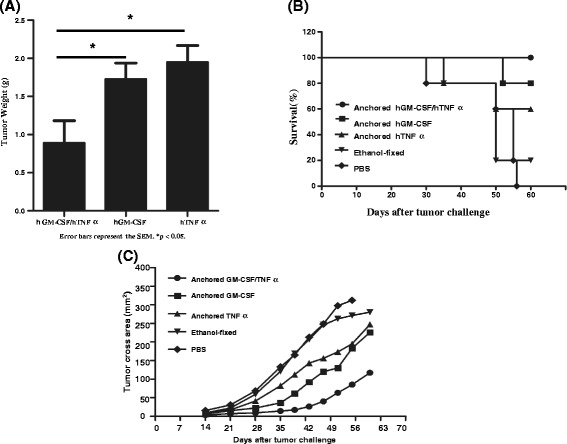
Therapeutic effects of hGM-CSF or/and hTNFα modified PC-3 cell vaccines on human prostate cancer in the huPBL-SCID mouse model. After inoculation with PC-3 prostate cancer cell vaccine, huPBL-SCID mice were treated i.p. with PC-3 cancer cell vaccines or PBS on days 0, 7, and 14. We collected and weighted the tumors in the mice died at different time points or those still living on day 60 (*p* < 0.05). There were no data at the time point on day 60 in the PBS control group because all mice died before/on day 56 (**a**). In addition, we drew a curve of the mean size of cross-sectional area of the tumors in each group along with the time period (**b**). Meanwhile, we recorded the time of all mice death with the day of injecting PC-3 cancer cells as the starting point and drew survival curves (*p* < 0.05) (**c**). The results represented one of three separate experiments

We tested therapeutic effects of different PC-3 cell vaccines modified with hGM-CSF and/or hTNFα on human prostate cancer in the huPBL-SCID mouse model. Compared with other cancer vaccines, the hGM-CSF/hTNFα doubly modified cancer vaccine significantly inhibited prostate cancer growth in terms of tumor weight (Fig. 1a) and size (Fig. 1b) and effectively prolonged the mice survival (Fig. 1c).

**Fig. 2:**
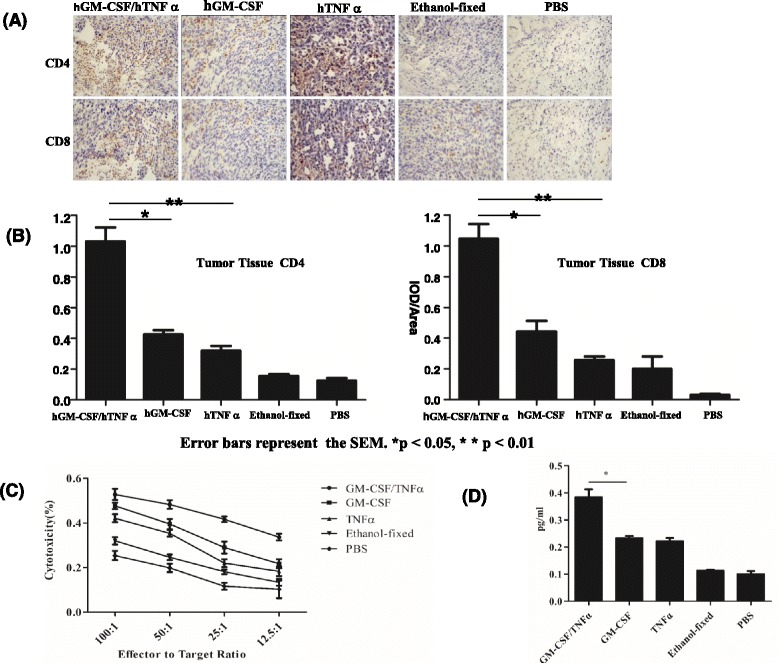
Immunohistochemical staining analysis of lymphocytes in tumor tissue and assessment of PC-3-specific cytotoxicity and IFNγ in spleen. Immunohistochemical staining analyzed CD4+ or CD8+ lymphocytes in the tumor tissues from huPBL-SCID mice 8 weeks after vaccination. The images of immunohistochemical staining were shown with ×200 magnification. Tumor tissues from different groups were stained with anti-hCD4 or anti-hCD8 antibody (**a**), and the quantitative analysis of the images was performed with integrated optic density (**b**). For PC-3-specific cytotoxicity assay, spleen cells were isolated on day 21 after tumor injection from each experimental group. Effector cells were stimulated by recombinant human IL-2 and mitomycin-treated PC-3 cancer cells. The supernatants were collected for the non-radioactive cytotoxicity assay (**c**). For quantification of IFNγ by ELISA, splenocytes were isolated from experimental mice 7 days after the last tumor vaccination and incubated with hIL-2 and mitomycin-treated PC-3 cancer cells for 48 h. The supernatants were collected for the measurement of IFNγ by ELISA (**d**). Error bars represented the SEM in both (**c**) and (**d**)

There were more human leukocytes and CD4+ or CD8+ lymphocytes residing in the lymph nodes in the hGM-CSF/hTNFα doubly modified group than in other groups 8 weeks after vaccination (Additional file 8). Similarly, more human CD4^+^ or CD8^+^ T lymphocytes were found to infiltrate into the tumor tissues in the hGM-CSF/hTNFα doubly modified group than in other groups (Fig. 2a, b), indicating that the hGM-CSF/hTNFα doubly modified PC-3 cell vaccine could enhance its anti-tumor immunity by increasing the infiltration of human T lymphocytes into the tumors.

We finally analyzed the tumor-specific cytotoxicity of cytotoxic T lymphocytes (CTL) in the spleen and revealed that the GM-CSF/TNFα doubly modified vaccine did establish a stronger tumor-specific T cell immunity than other PC-3 cell vaccines (Fig. 2c). The supernatant from in vitro splenocyte culture in the GM-CSF/TNFα doubly modified group had the highest production of PC-3-specific IFNγ (Fig. 2d). Both results indicated that type 1 protective immunity was induced against the human prostate cancer.

Therefore, our current study provided a solid foundation for potential clinical application of this novel hGM-CSF/hTNFα surface-modified prostate cancer therapeutic vaccine. This unique approach can be easily adopted to generate a personalized whole cancer cell vaccine from individual autologous cancer cells, thereby potentially overcoming cancer antigen heterogeneity [9–11].

## Additional files

Additional file 1:
**Materials and methods.**
Additional file 2:
**Flow cytometric analysis of the modification efficiency of streptavidin (SA)-tagged hGM-CSF and/or hTNFα on the surface of ethanol-fixed and biotinylated PC-3 prostate cancer cell vaccine.** Ethanol-fixed PC-3 cells were biotinylated and then incubated with SA-hGM-CSF, SA-hTNFα, or both as described before. The presence of hGM-CSF or/and hTNFα was assessed with allophycocyanin (APC)-labeled anti-hGM-CSF (1:400) or/and fluorescein isothiocyanate (FITC)-labeled anti-hTNFα (1:100) monoclonal antibodies for flow cytometric analysis. Ethanol-fixed PC-3 cells were used as control. Additional file 3:
**Bioactive assays of hGM-CSF and hTNFα immobilized on the surface of ethanol-fixed PC-3 prostate cancer cell vaccine.** Membrane fractions were prepared from the hGM-CSF/hTNFα doubly modified PC-3 cancer cell vaccine as described in materials and methods. (A) Bioactive assay of hGM-CSF immobilized on the surface of ethanol-fixed PC-3 cell vaccine. The proliferative activity of membrane-conjugated hGM-CSF was assessed on TF-1 cells with SA-hGM-CSF/SA-hTNFα un-modified PC-3 cell vaccine as negative control. (B) Bioactive assay of hTNF-α immobilized on the surface of ethanol-fixed PC-3 cell vaccine. The cytotoxic activity of membrane-conjugated hTNFα was assessed on L929 cells with hGM-CSF/hTNFα un-modified PC-3 cell vaccine as negative control. Data were shown as the mean ± SEM of triplicates. Additional file 4:
**Flow cytometric analysis of tumor tissue and peripheral blood from NOD/SCID mice with anti-hPSMA and anti-HLA.** Tumor tissues and peripheral blood were isolated on the 30th and 60th day to prepare single cell suspensions. The presence of PSMA and HLA (human leukocyte antigen) was assessed with APC-labeled anti-hPSMA (1:100) and FITC-labeled anti-hHLA (1:200) monoclonal antibodies, respectively, for flow cytometric analysis. The LNCaP prostate cancer cells were used as a positive control because they could secrete PSA and express PSMA. (**A**) The single cell suspension from tumor tissue on day 30 with APC-labeled anti-hPSMA. (**B**) The LNCaP prostate cancer cells. (**C**) The single cell suspension from tumor tissue on day 30 with FITC-labeled anti-hHLA. (**D**) The blood from PC-3 inoculated NOD/SCID mice on day 30 with FITC-labeled anti-hHLA. (**E**) The white cells from PC-3 inoculated NOD/SCID mice on day 60 with FITC-labeled anti-hHLA. Additional file 5:
**Screen for the immune “leakage” phenomenon of NOD/SCID mice and detection of the levels of NK cells before and after injection of NK cell inhibitor GM1.** (**A**) Concentration of IgG in peripheral blood serum of NOD/SCID mice. The immune “leakage” phenomenon was screened by the levels of IgG through ELISA kit (Beyotime) in 25 NOD/SCID mice with standard and blank controls (data from the controls not shown). The concentration of IgG lower than 10 μg/ml was defined as no immune leakage. (**B**) The changes of CD122 levels in peripheral blood of NOD/SCID mice before and after injection of NK cell inhibitor anti-asialo-GM1 antibody. CD122 was assessed with FITC-labeled anti-CD122 (1:200) monoclonal antibody for flow cytometric analysis. Left: CD122 level before injection of NK cell inhibitor anti-asialo-GM1 antibody; middle: CD122 level after injection of NK cell inhibitor anti-asialo-GM1 antibody; right: comparison of CD122 levels before and after injection of NK cell inhibitor anti-asialo-GM1 antibody. Additional file 6:
**Flow cytometric analysis of human lymphocytes in the peripheral blood and spleen tissue of huPBL-SCID mice after huPBL transplantation.** (**A**) The presence of CD45^+^, CD8^+^, or CD4^+^ cells was assessed respectively with phycoerythrin (PE)-labeled anti-hCD45, PE-Cy7-labeled anti-hCD8, and PE-Cy7-labeled anti-hCD4 monoclonal antibodies for flow cytometric analyses. (**B**) Flow cytometric analysis of human CD45^+^ cells in the spleen tissue of huPBL-SCID mice 8 weeks after huPBL transplantation. Spleen was isolated from huPBL-SCID 8 weeks after huPBL transplantation and single cell suspensions were prepared from the isolated spleen. The presence of CD45^+^ cells was assessed with PE-labeled anti-hCD45 monoclonal antibody through a flow cytometer. NOD/SCID mice without huPBL transplantation were used as the negative controls. Additional file 7:
**Immunohistochemical staining of spleen and liver tissues from huPBL-SCID mice 8 weeks after huPBL transplantation.** The images of immunohistochemical staining were shown with ×200 magnification. The spleen and liver tissues from huPBL-SCID mice were stained with anti-hCD4 or anti-hCD8 antibody (**A**), and the quantitative analysis of the images was performed with integrated optic density (**B**). The spleen and liver tissues from NOD/SCID mice without huPBL transplantation were used as negative controls. Additional file 8:
**Immunohistochemical staining analysis of the lymph node tissues from huPBL-SCID mice 8 weeks after vaccination.** The images of immunohistochemical staining were shown with ×200 magnification. Lymph node tissues from different groups were stained with rat anti-hCD45 (**A**), anti-hCD4 (**B**), or anti-hCD8 antibody (**C**). The quantitative analysis of those images was carried out with integrated optic density (**D**).

## Abbreviations

hGM-CS: human granulocyte-macrophage colony-stimulating factor
hTNFα: human tumor necrosis factor α
SA: streptavidin
huPBL: human peripheral blood lymphocytes
IFN: interferon
NOD/SCID: nonobese diabetic/severe combined immunodeficiency
PSA: prostate-specific antigen
PSMA: prostate-specific membrane antigen
NK: natural killer
PBS: phosphate-buffered saline
ELISA: enzyme-linked immunosorbent assay
FITC: fluorescein isothiocyanate
HLA: human leukocyte antigen
APC: allophycocyanin
PE: phycoerythrin

## References

[1] Simons JW (2014). Prostate cancer immunotherapy: beyond immunity to curability. Cancer Immunol Res..

[2] Shi L, Chen S, Yang L, Li Y (2013). The role of PD-1 and PD-L1 in T-cell immune suppression in patients with hematological malignancies. J Hematol Oncol..

[3] Han E, Li X, Wang C, Li T, Han S (2013). Chimeric antigen receptor-engineered T cells for cancer immunotherapy: progress and challenges. J Hematol Oncol..

[4] Gao J, Huang S, Li M, Luo R, Wang X, Takashima A (2006). GM-CSF-surface-modified B16.F10 melanoma cell vaccine. Vaccine.

[5] Hu Z, Tan W, Zhang L, Liang Z, Xu C, Su H (2010). A novel immunotherapy for superficial bladder cancer by intravesical immobilization of GM-CSF. J Cell Mol Med..

[6] Zhang X, Shi X, Li J, Hu Z, Zhou D, Gao J (2012). A novel therapeutic vaccine of mouse GM-CSF surface modified MB49 cells against metastatic bladder cancer. J Urol..

[7] Yin W, He Q, Hu Z, Chen Z, Mao Q, Sun Z (2010). A novel therapeutic vaccine of GM-CSF/TNFalpha surface-modified RM-1 cells against the orthotopic prostatic cancer. Vaccine..

[8] Bankert RB, Hess SD, Egilmez NK (2002). SCID mouse models to study human cancer pathogenesis and approaches to therapy: potential, limitations, and future directions. Front Biosci..

[9] Boyd LK, Mao X, Lu YJ (2012). The complexity of prostate cancer: genomic alterations and heterogeneity. Nat Rev Urol..

[10] Spans L, Clinckemalie L, Helsen C, Vanderschueren D, Boonen S, Lerut E (2013). The genomic landscape of prostate cancer. Int J Mol Sci..

[11] Wyatt AW, Mo F, Wang Y, Collins CC (2013). The diverse heterogeneity of molecular alterations in prostate cancer identified through next-generation sequencing. Asian J Androl..

