# Supplementary material for: Evaluation of hGM-CSF/hTNFα surface-modified prostate cancer therapeutic vaccine in the huPBL-SCID chimeric mouse model
Source: J Hematol Oncol. 2015 Jun 25;8:76. doi: 10.1186/s13045-015-0175-8 (PMC4490636; doi:10.1186/s13045-015-0175-8)
Supplement: Additional file 1: — Materials and methods. [file 13045_2015_175_MOESM1_ESM.docx]

**Materials and Methods**

**1 Cell lines, mice and reagents**

The prostate cancer cell lines PC-3 and LNCaP as well as TF-1 and L929 cell lines were purchased from the cell bank of Chinese Academy of Sciences (Beijing, China), and cultured in F12 medium containing 2% FBS (fetal bovine serum) in 37°C humidified incubator with 5%CO_2_. NOD-SCID male mice of approximately 4-6 weeks old were purchased from Vital River Laboratory Animal Technology (Beijing, China). All animal studies were carried out in accordance with the guidelines of Wenzhou Medical University and approved by Ethics Committee for Animal Scientific Procedures of Wenzhou Medical University(Permit No: wydw2011-0010 to Dr. Gao). SA-hGM-CSF and SA-hTNFα bi-functional fusion proteins were prepared in our laboratory [4-7].

**2 Vaccine preparation**

PC-3 prostate cancer cells were fixed in 30% ethanol (v/v) for 1 h at room temperature and their viability was assessed by trypan blue (0.4%) staining. Ethanol-fixed PC-3 cells (2×10^7^cells/ml) were incubated with 10 mM fresh EZ-Link Sulfo-NHS-LC-biotin for 30 min at room temperature, and washed three times with 1xPBS (phosphate-buffered saline) containing 100 mM glycine. The biotinylated cells were incubated for 1 h with SA-hGM-CSF, SA-hTNFα or both at 200 ng/10^6^ cells, and then washed three times with 1xPBS. The presence of hGM-CSF or/and hTNF α immobilized on the cell surface was assessed by flow cytometry (BD, USA).

**3 Bioactive assay of hGM-CSF or hTNF**α **immobilized on the surface of ethanol-fixed PC-3 prostate cancer cells**

1×10^5^ ethanol-fixed PC-3 cells modified by hGM-CSF or/and hTNFα were pelleted and re-suspended in 0.5 ml of 1xPBS. The cells were lysed by three cycles of freeze and thaw. Membrane fractions were harvested by centrifugation at 15,000×g at 4°C for 5 min and re-suspended in 0.5 ml of F12 medium containing 2% FBS.

As described before, the bioactivity of hGM-CSF was assessed by TF-1 cell proliferation assay with un-biotinylated PC-3 cells which were pre-incubated with SA-hGM-CSF as a negative control [4-7]. Briefly, aliquots of the cell membrane fractions as indicated were added to 1×10^4^ TF-1 cells/each well, and cultured for 3 days at 37 °C in a 5%CO_2_ humidified incubator. WST-8 (Cell Counting Kit-8, Beyotime) with the final concentration 10% was added to each well and incubated further for 5 h, and the absorbance was read at 450 nm on a microplate reader.

hTNFα bioactivity was assessed by its cytotoxicity on L929 fibroblast cells as described before [7]. Briefly, L929 cells were plated in triplicates at 5×10^6^/ml each well in the presence of actinomycin D (1μg/ml), and incubated with indicated aliquots of the cell membrane fractions prepared above. After incubation for 20 h, WST-8 (Cell Counting Kit-8) from Beyotime (Beijing, China) with the final concentration 10% was added and incubated further for 4 h, and the absorbance was read at 450 nm on a microplate reader.

**4 Detection of the “leakage phenomenon” in NOD/SCID mice**

Approximately 100ul peripheral blood of NOD/SCID mice were collected from mouse tail, following with addition of EDTA (ethylenediaminetetraacetic acid) as anti-coagulant. Peripheral blood serum was harvested by centrifugation at 3000 rpm for 20-30 min. IgG concentrations of NOD/SCID mice from peripheral blood serum were assessed by ELISA (enzyme-linked immunosorbent assay). Lower than 36 ug/ml of IgG concentration in peripheral blood serum from all NOD/SCID mice was defined as “no phenomenon of immune leakage” [8].

**5 Immunologic reconstitution in NOD/SCID mice**

NOD/SCID mice without immune leakage were used to proceed for immunological reconstitution. NK cell inhibitor, Anti Asialo GM1 antibody (Rabbit), was injected through tail vein about 20μl every time. The efficiency was reduced gradually over 4 days after the first injection, and 3 times of injection were needed in the following 2 weeks. Separated human peripheral blood lymphocytes (huPBL) were injected (i.p.) into each mouse at 4×10^7^ cells to build the huPBL-SCID mice. Four weeks after huPBL transfer, CD4^+^, CD8^+^, and CD45^+^ cells could be detected in peripheral blood of the mice regularly. The mice were closely observed for mental status, motility, reaction and [nutritional status](javascript:showjdsw('jd_t','j_')), etc., and further evaluated whether the phenomenon of GVHD existed.

**6 Establishment of the model of human prostate cancer and cancer vaccination**

huPBL-SCID mice were inoculated subcutaneously with 5×10^5^ PC-3 prostate cancer cells in 20ul 1xPBS suspension into right flanks after anesthetized by 0.6% pentobarbital sodium. The grouped mice were injected intraperitoneally with 1xPBS or different 2×10^6^ cancer cell vaccines on the same day (day 0). The booster vaccination of those therapeutic prostate cancer vaccines was repeated on days 7 and 14. The mice were sacrificed on day 60 and the tumors were collected and weighted. The survival of the mice was continuously monitored for sixty days. The experiments were repeated three times.

**7 Tumor-specific cytotoxicity assay**

Splenocytes were isolated from each group on day 21, and then stimulated in vitro with 20 U/ml of recombinant hIL-2 (R & D systems, Minneapolis, MN) plus mitomycin-treated PC-3 cells for 5 days. Effector cells were harvested by Ficoll-Hypaque density centrifugation and adjusted at 1 × 10^5^ cells/ml in the F12 medium. Target PC-3 cells were seeded in triplicate at 1 × 10^4^ cells in the 100 µl medium per well in 96-well plates and a various number of effector cells was added to the target PC-3 cells at the desired E:T ratios. After culturing for 4 h at 37°C, the supernatant was collected to measure lactate dehydrogenase (LDH) activity by CytoTox 96 non-radioactive cytotoxicity assay (Promega, Madison, WI). Percentage of cytotoxicity was calculated as follows:

$$\%cytotoxicity=100\times\frac{(Experimental-effector \text{s}pontaneous-target spontaneous)}{(target maximum-target spontaneous)}$$

**8 Determination of IFNγ production by ELISA**

Splenocytes were isolated from experimental mice on day 21, and then stimulated in vitro with 20 U/ml of recombinant hIL-2 (R & D systems) plus mitomycin-treated PC-3 cancer cells. Culture supernatants were harvested, and IFNγ production was measured by ELISA (R & D Systems).

**9 Immunohistochemical (IHC) analysis**

The spleen and liver tissues from experimental mice were collected from huPBL-SCID mice 8 weeks after transfer of huPBL and submitted for IHC examinations. The tumor and lymph node tissues from experimental mice were obtained from huPBL-SCID mice on day 60 (except those from the PBS group on day 56). Briefly, the spleen, liver, lymph node, and tumor sections were incubated with rat anti-CD45 (1:200), anti-hCD4 (1:200) or anti-hCD8 (1:200) (Abcam, Cambridge, MA) overnight at 4°C and washed with 1xPBS. Then, they were incubated with anti-rat HRP-IgG antibody and counter-stained with haematoxylin and eosin (HE). IHC images were quantified by the Image pro plus software with integrated optic density (IOD).

**10 Statistical analysis**

Graphpad Prism 5 and SPSS17.0 softwares were used for statistical analysis. *P*<0.05 was considered as significant.
