# Supplementary figures and images for: Evaluation of hGM-CSF/hTNFα surface-modified prostate cancer therapeutic vaccine in the huPBL-SCID chimeric mouse model
Source: J Hematol Oncol. 2015 Jun 25;8:76. doi: 10.1186/s13045-015-0175-8 (PMC4490636; doi:10.1186/s13045-015-0175-8)

## Slide 1
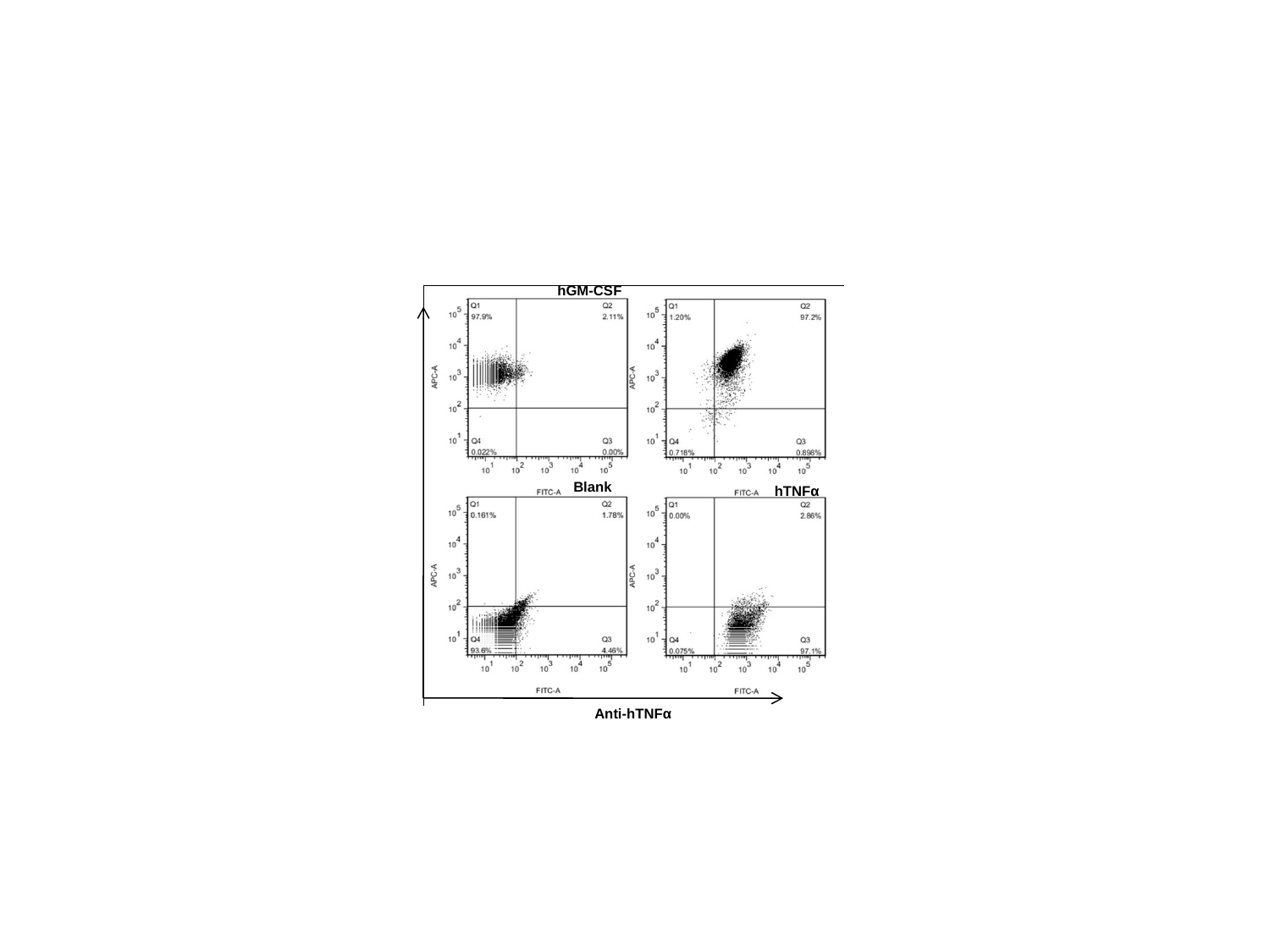

hGM-CSF
Blank
hTNFα
Anti-hTNFα

Supplement: Additional file 2: — Flow cytometric analysis of the modification efficiency of streptavidin (SA)-tagged hGM-CSF and/or hTNFα on the surface of ethanol-fixed and biotinylated PC-3 prostate cancer cell vaccine. Ethanol-fixed PC-3 cells were biotinylated and then incubated with SA-hGM-CSF, SA-hTNFα, or both as described before. The presence of hGM-CSF or/and hTNFα was assessed with allophycocyanin (APC)-labeled anti-hGM-CSF (1:400) or/and fluorescein isothiocyanate (FITC)-labeled anti-hTNFα (1:100) monoclonal antibodies for flow cytometric analysis. Ethanol-fixed PC-3 cells were used as control. [file 13045_2015_175_MOESM2_ESM.ppt]

## Slide 1
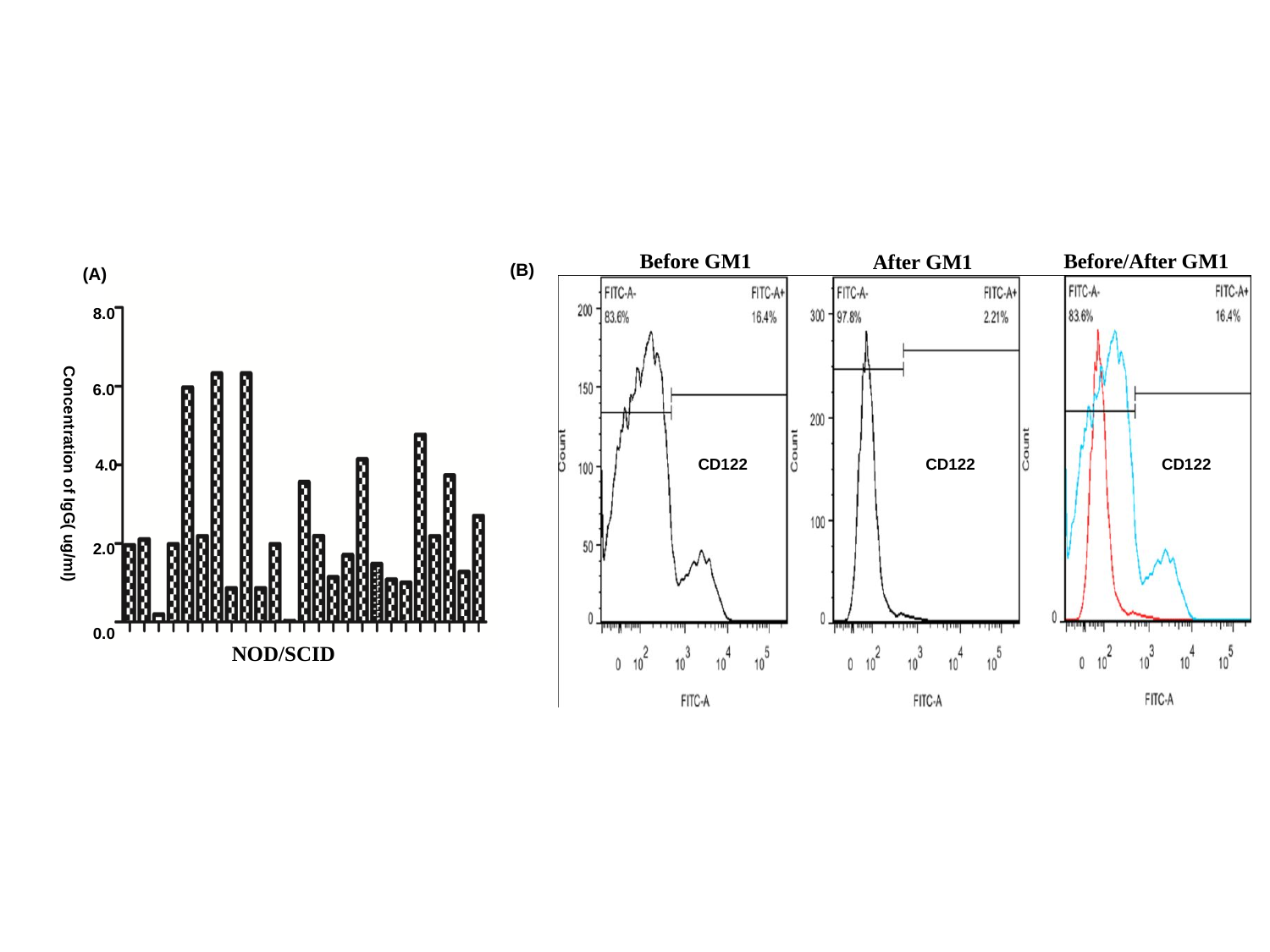

Before GM1
 After GM1
Before/After GM1
CD122
CD122
CD122
(B)
(A)
8.0
 Concentration of IgG( ug/ml)
6.0
4.0
2.0
0.0
NOD/SCID

Supplement: Additional file 5: — Screen for the immune “leakage” phenomenon of NOD/SCID mice and detection of the levels of NK cells before and after injection of NK cell inhibitor GM1. (A) Concentration of IgG in peripheral blood serum of NOD/SCID mice. The immune “leakage” phenomenon was screened by the levels of IgG through ELISA kit (Beyotime) in 25 NOD/SCID mice with standard and blank controls (data from the controls not shown). The concentration of IgG lower than 10 μg/ml was defined as no immune leakage. (B) The changes of CD122 levels in peripheral blood of NOD/SCID mice before and after injection of NK cell inhibitor anti-asialo-GM1 antibody. CD122 was assessed with FITC-labeled anti-CD122 (1:200) monoclonal antibody for flow cytometric analysis. Left: CD122 level before injection of NK cell inhibitor anti-asialo-GM1 antibody; middle: CD122 level after injection of NK cell inhibitor anti-asialo-GM1 antibody; right: comparison of CD122 levels before and after injection of NK cell inhibitor anti-asialo-GM1 antibody. [file 13045_2015_175_MOESM5_ESM.ppt]

## Slide 1
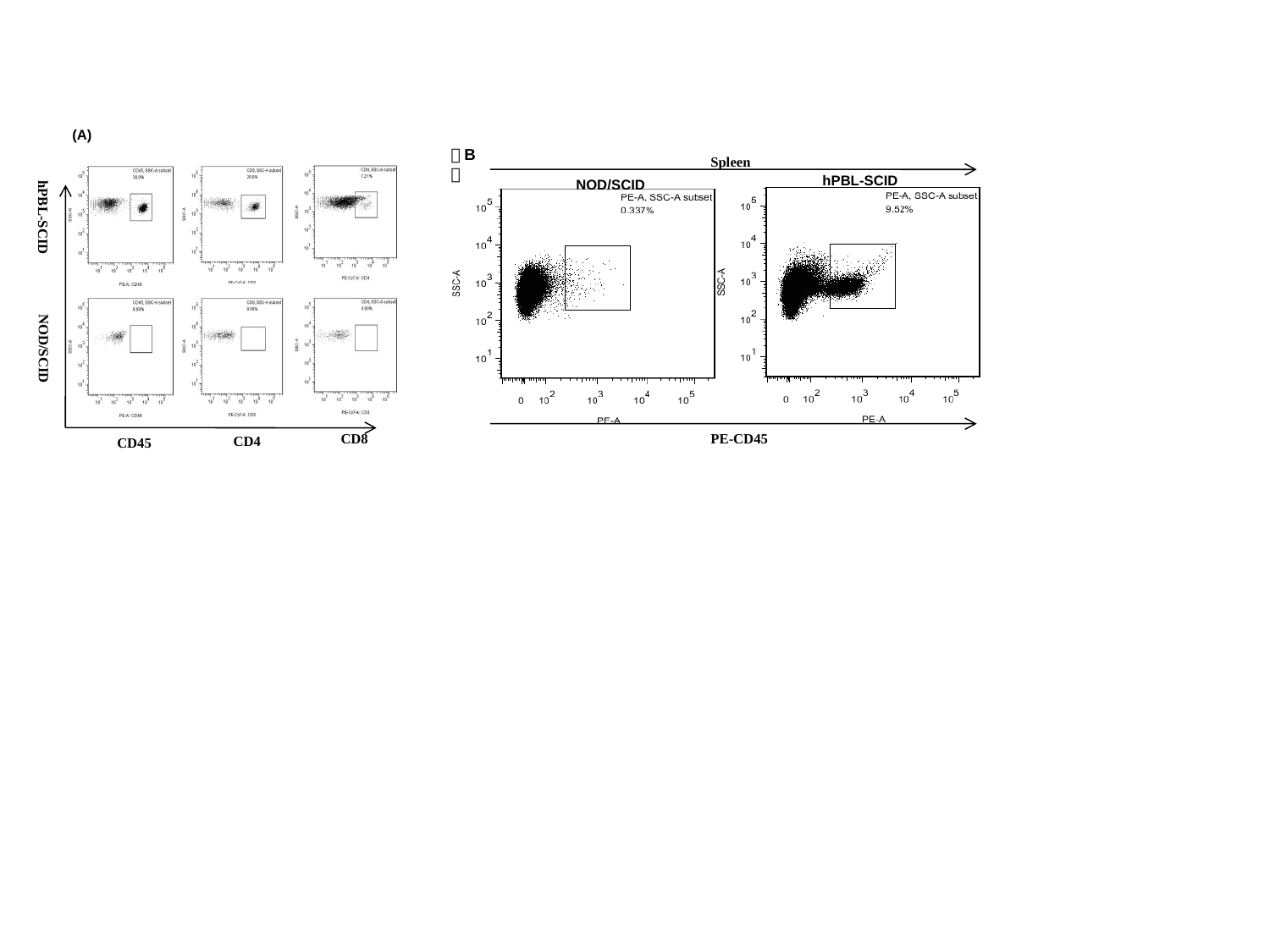

(A)
	（B）
Spleen
hPBL-SCID
NOD/SCID
PE-CD45
hPBL-SCID
NOD/SCID
CD8
CD4
CD45

Supplement: Additional file 6: — Flow cytometric analysis of human lymphocytes in the peripheral blood and spleen tissue of huPBL-SCID mice after huPBL transplantation. (A) The presence of CD45+, CD8+, or CD4+ cells was assessed respectively with phycoerythrin (PE)-labeled anti-hCD45, PE-Cy7-labeled anti-hCD8, and PE-Cy7-labeled anti-hCD4 monoclonal antibodies for flow cytometric analyses. (B) Flow cytometric analysis of human CD45+ cells in the spleen tissue of huPBL-SCID mice 8 weeks after huPBL transplantation. Spleen was isolated from huPBL-SCID 8 weeks after huPBL transplantation and single cell suspensions were prepared from the isolated spleen. The presence of CD45+ cells was assessed with PE-labeled anti-hCD45 monoclonal antibody through a flow cytometer. NOD/SCID mice without huPBL transplantation were used as the negative controls. [file 13045_2015_175_MOESM6_ESM.ppt]
