# Supplementary material for: Evaluation of hGM-CSF/hTNFα surface-modified prostate cancer therapeutic vaccine in the huPBL-SCID chimeric mouse model
Source: J Hematol Oncol. 2015 Jun 25;8:76. doi: 10.1186/s13045-015-0175-8 (PMC4490636; doi:10.1186/s13045-015-0175-8)
Supplement: Additional file 3: — Bioactive assays of hGM-CSF and hTNFα immobilized on the surface of ethanol-fixed PC-3 prostate cancer cell vaccine. Membrane fractions were prepared from the hGM-CSF/hTNFα doubly modified PC-3 cancer cell vaccine as described in materials and methods. (A) Bioactive assay of hGM-CSF immobilized on the surface of ethanol-fixed PC-3 cell vaccine. The proliferative activity of membrane-conjugated hGM-CSF was assessed on TF-1 cells with SA-hGM-CSF/SA-hTNFα un-modified PC-3 cell vaccine as negative control. (B) Bioactive assay of hTNF-α immobilized on the surface of ethanol-fixed PC-3 cell vaccine. The cytotoxic activity of membrane-conjugated hTNFα was assessed on L929 cells with hGM-CSF/hTNFα un-modified PC-3 cell vaccine as negative control. Data were shown as the mean ± SEM of triplicates. [file 13045_2015_175_MOESM3_ESM.ppt]

## Slide 1
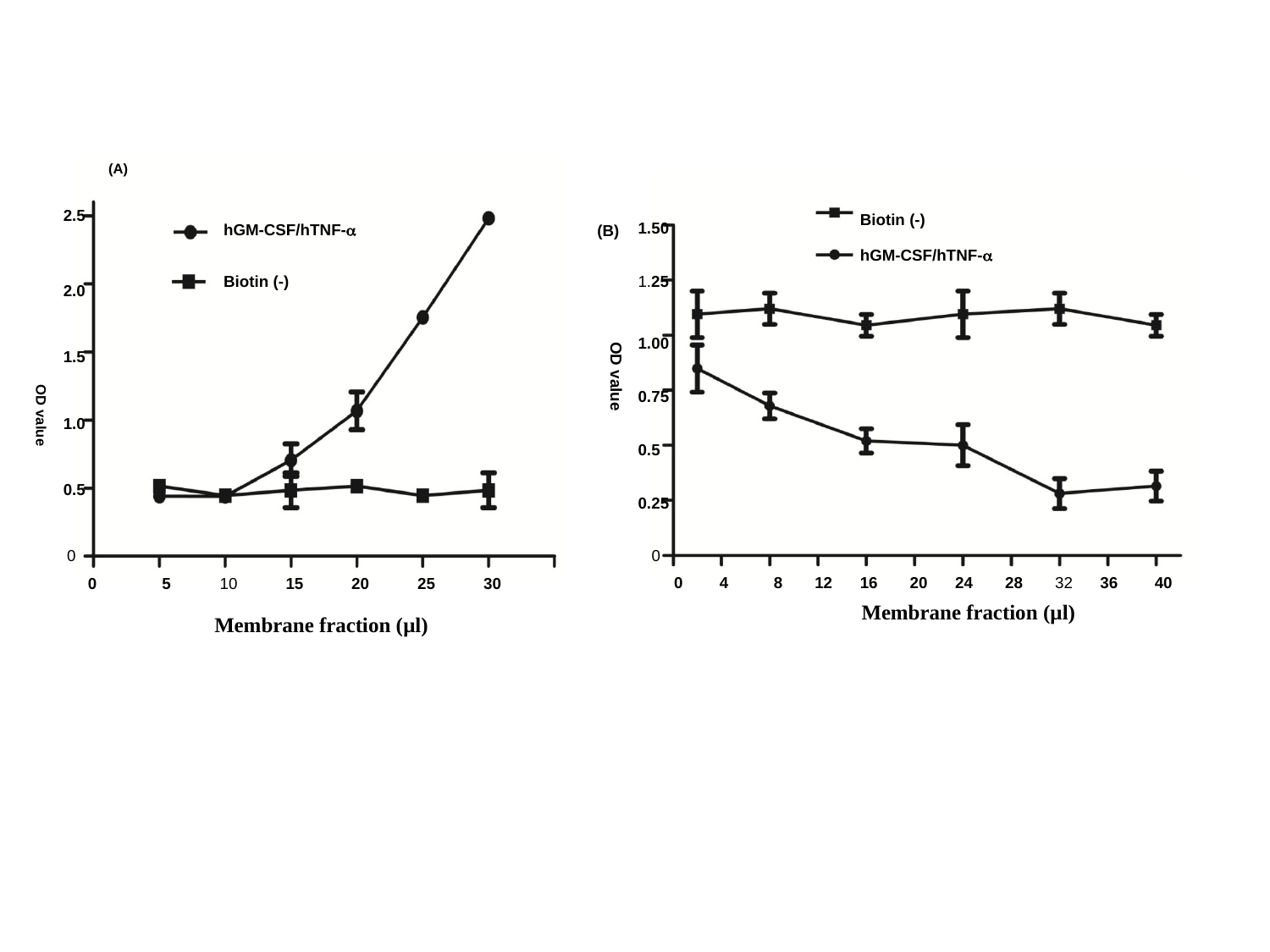

2.5
hGM-CSF/hTNF-α
Biotin (-)
 2.0
 1.5
OD value
 1.0
 0.5
0
 0
 5
 10
 15
 20
 25
 30
Membrane fraction (μl)
(A)
	(B)
Biotin (-)
 1.50
hGM-CSF/hTNF-α
 1.25
 1.00
OD value
 0.75
 0.5
 0.25
0
 0
 4
 8
12
16
 20
 24
28
 32
 36
 40
Membrane fraction (μl)
