# Supplementary material for: Evaluation of hGM-CSF/hTNFα surface-modified prostate cancer therapeutic vaccine in the huPBL-SCID chimeric mouse model
Source: J Hematol Oncol. 2015 Jun 25;8:76. doi: 10.1186/s13045-015-0175-8 (PMC4490636; doi:10.1186/s13045-015-0175-8)
Supplement: Additional file 4: — Flow cytometric analysis of tumor tissue and peripheral blood from NOD/SCID mice with anti-hPSMA and anti-HLA. Tumor tissues and peripheral blood were isolated on the 30th and 60th day to prepare single cell suspensions. The presence of PSMA and HLA (human leukocyte antigen) was assessed with APC-labeled anti-hPSMA (1:100) and FITC-labeled anti-hHLA (1:200) monoclonal antibodies, respectively, for flow cytometric analysis. The LNCaP prostate cancer cells were used as a positive control because they could secrete PSA and express PSMA. (A) The single cell suspension from tumor tissue on day 30 with APC-labeled anti-hPSMA. (B) The LNCaP prostate cancer cells. (C) The single cell suspension from tumor tissue on day 30 with FITC-labeled anti-hHLA. (D) The blood from PC-3 inoculated NOD/SCID mice on day 30 with FITC-labeled anti-hHLA. (E) The white cells from PC-3 inoculated NOD/SCID mice on day 60 with FITC-labeled anti-hHLA. [file 13045_2015_175_MOESM4_ESM.ppt]

## Slide 1
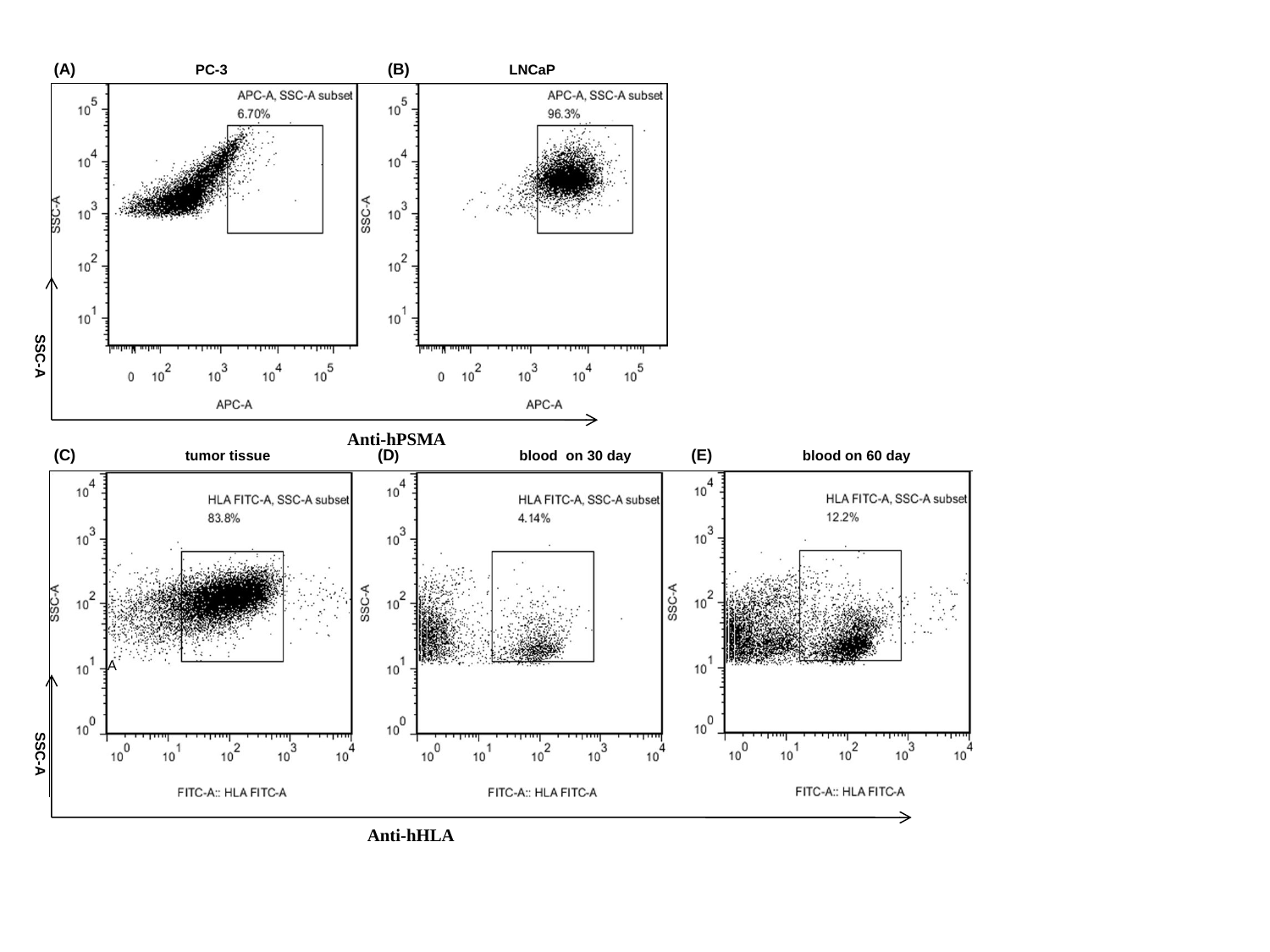

(A)
(B)
PC-3
LNCaP
SSC-A
Anti-hPSMA
(C)
(D)
(E)
tumor tissue
blood on 30 day
blood on 60 day
blood 60 day
A
SSC-A
Anti-hHLA
