# Supplementary material for: Evaluation of hGM-CSF/hTNFα surface-modified prostate cancer therapeutic vaccine in the huPBL-SCID chimeric mouse model
Source: J Hematol Oncol. 2015 Jun 25;8:76. doi: 10.1186/s13045-015-0175-8 (PMC4490636; doi:10.1186/s13045-015-0175-8)
Supplement: Additional file 7: — Immunohistochemical staining of spleen and liver tissues from huPBL-SCID mice 8 weeks after huPBL transplantation. The images of immunohistochemical staining were shown with ×200 magnification. The spleen and liver tissues from huPBL-SCID mice were stained with anti-hCD4 or anti-hCD8 antibody (A), and the quantitative analysis of the images was performed with integrated optic density (B). The spleen and liver tissues from NOD/SCID mice without huPBL transplantation were used as negative controls. [file 13045_2015_175_MOESM7_ESM.ppt]

## Slide 1
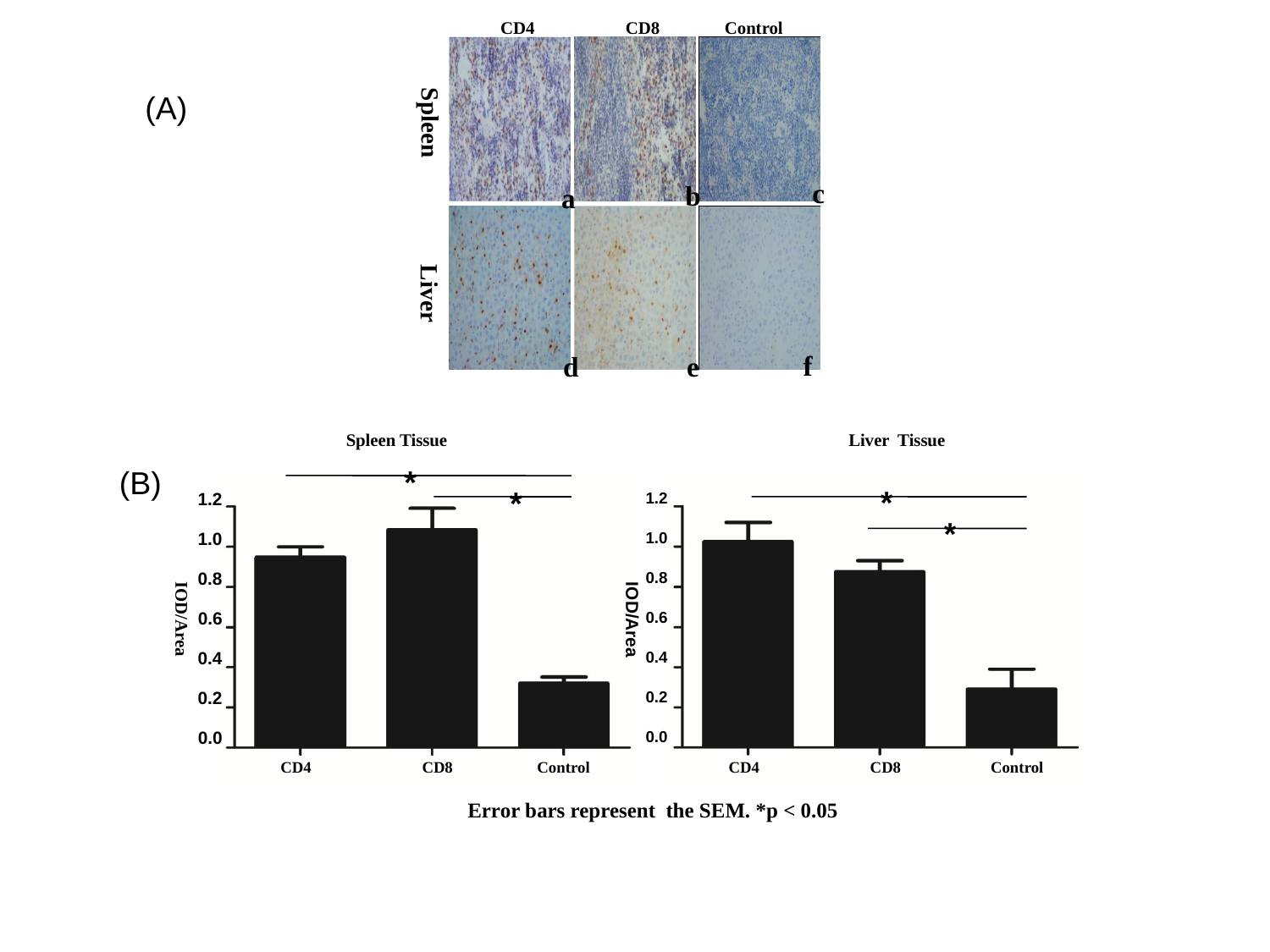

CD8
CD4
Control
Spleen
	a
	b
c
 Liver
	f
	d
	e
(A)
Spleen Tissue
Liver Tissue
*
*
*
 1.2
 1.2
*
 1.0
 1.0
 0.8
 0.8
 IOD/Area
 IOD/Area
 0.6
 0.6
 0.4
 0.4
 0.2
 0.2
 0.0
 0.0
CD8
Control
CD4
 Control
CD4
CD8
Error bars represent the SEM. *p < 0.05
(B)
