# Supplementary material for: Evaluation of hGM-CSF/hTNFα surface-modified prostate cancer therapeutic vaccine in the huPBL-SCID chimeric mouse model
Source: J Hematol Oncol. 2015 Jun 25;8:76. doi: 10.1186/s13045-015-0175-8 (PMC4490636; doi:10.1186/s13045-015-0175-8)
Supplement: Additional file 8: — Immunohistochemical staining analysis of the lymph node tissues from huPBL-SCID mice 8 weeks after vaccination. The images of immunohistochemical staining were shown with ×200 magnification. Lymph node tissues from different groups were stained with rat anti-hCD45 (A), anti-hCD4 (B), or anti-hCD8 antibody (C). The quantitative analysis of those images was carried out with integrated optic density (D). [file 13045_2015_175_MOESM8_ESM.ppt]

## Slide 1
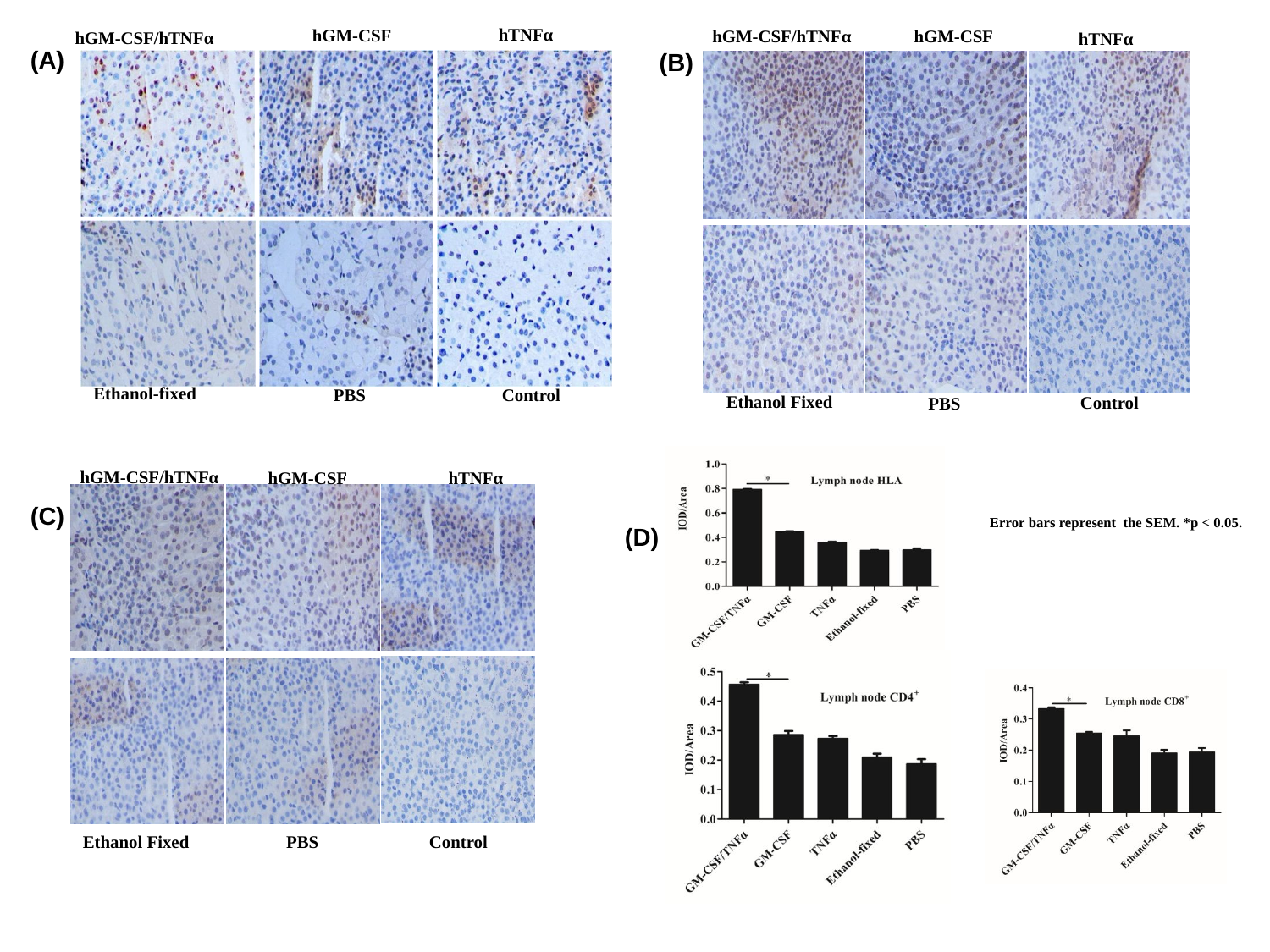

hTNFα
hGM-CSF
hGM-CSF/hTNFα
Ethanol-fixed
 Control
PBS
hGM-CSF/hTNFα
hGM-CSF
hTNFα
Ethanol Fixed
Control
PBS
(A)
(B)
hGM-CSF/hTNFα
hGM-CSF
hTNFα
Ethanol Fixed
PBS
Control
(C)
Error bars represent the SEM. *p < 0.05.
(D)
